# The Law Relating to Medical Officers of Health (Outside Metropolis).—V

**Published:** 1903-08-29

**Authors:** J. E. R. Stephens

**Affiliations:** Barrister-at-Law, etc.


### HOSPITAL ADMINISTRATION.

### CONSTRUCTION AND ECONOMICS.

### THE LAW RELATING TO MEDICAL OFFICERS OF HEALTH (OUTSIDE METROPOLIS).—V.

By J. E. R. Stephens, Barrister-at-Law, etc.

(*Concluded from page* 285.)

Under Factory and Workshop Act, 1901.

It is provided by section 2, sub-section 3, that where, on the certificate of a medical officer of health or inspector of nuisances, it appears to any district council that the limewashing, cleansing, or purifying of any such workshop, or of any part thereof, is necessary for the health of the persons employed therein, the council shall give notice in writing to the owner or occupier of the workshop to limewash, cleanse, or purify the same, or part thereof, as the case may require. If the person to whom notice is so given fails to comply therewith within the time therein specified, he shall be liable to a fine not exceeding 10s. for every day during which he continues to make default, and the council may, if they think fit, cause the workshop or part to be limewashed, cleansed, or purified, and may recover in a summary manner the expenses incurred by them in so doing from the person in default. This section shall not apply to any workshop or workplace to which the Public Health (London) Act, 1891, applies.

By section 5, where it appears to a factory inspector that any act, neglect, or default, in relation to any drain, water-closet, earth-closet, privy, ashpit, water-supply, nuisance or other matter in a factory or workshop, is punishable or remediable under the law relating to public health, but not under this Act, that inspector shall give notice in writing of the act, neglect, or default, to the district council in whose district the factory or workshop is situate, and it shall be the duty of the district council to make such inquiry into the subject of the notice, and to take such action thereon, as seems to that council proper for the purpose of enforcing the law, and to inform the inspector of the proceedings taken in consequence of the notice. An inspector may, for the purposes of this section, take with him into a factory or a workshop a medical officer of health, inspector of nuisances, or other officer of the district council. This section provides a remedy for sanitary defects, etc., in those factories and workshops which do not come within the operation of this Act, and which are therefore subject to the general law relating to public health and within the jurisdiction of district councils, and specifically gives to an inspector power which he has not otherwise. This section does not override the right of a council acting on the report of their surveyor under section 38 of the Public Health Act, 1875, to require the owner or occupier of a house used as a factory in which persons of both sexes are employed to construct sufficient privy accommodation, etc., for the separate use of each sex, and which renders any such owner or occupier neglecting or refusing to comply with such order liable to a penalty not exceeding £20, and a further fine of 40s. for every day during which the default is continued.

*Bakehouses.*—Section 102 of the Factory Act, 1901, enacts that as respects every retail bakehouse the provisions of this part of this Act shall be enforced by the district council of the district in which the retail bakehouse is situate, and not by an inspector; and for the purposes of this section the medical officer of health of the district council shall have and may exercise all the powers of entry, inspection, taking legal proceedings and otherwise of an inspector. In this section the expression "retail bakehouse" means any bakehouse or place, not being a factory, the bread, biscuits, or confectionery baked in which are sold, not wholesale, but by retail, in some shop or place occupied with the bakehouse. The words "this part of this Act" apparently refer to sections 97 to 102, which contain the provisions relating to bakehouses. District councils are to enforce the provisions of these sections with regard to retail bakehouses, and not factory inspectors.

Section 97 provides that a place shall not be used as a bakehouse unless the following regulations are complied with:—

(*a*) A water-closet, earth-closet, privy, or ashpit must not be within or communicate directly with the bakehouse.

(*b*) Every cistern for supplying water to the bakehouse must be separate and distinct from any cistern for supplying water to a water-closet.

(*c*) A drain or pipe for carrying off fæcal or sewage matter must not have an opening within the bakehouse. If any person lets or suffers to be occupied or occupies any room or place as a bakehouse in contravention of this section, he shall be liable to a fine not exceeding 40s., and to a further fine not exceeding 5s. for every day during which any room or place is so occupied after a conviction under this section.

Section 99 enacts that all the inside walls of the rooms of a bakehouse, and all the ceilings or tops of those rooms (whether those walls, ceilings, or tops are plastered or not), and all the passages and staircases of a bakehouse, must either be painted with oil or varnished or be limewashed, or be partly painted or varnished and partly limewashed; and

(*a*) Where the bakehouse is painted with oil or varnished, there must be three coats of paint or varnish, and the paint or varnish must be renewed once at least in every seven years, and must be washed with hot water and soap once at least in every six months; and

(*b*) Where the bakehouse is limewashed, the limewashing must be renewed once, at least, in every six months.

Under section 100, a place on the same level with a bakehouse, and forming part of the same building, may not be used as a sleeping-place, unless it is constructed as follows, that is to say

(*a*) Is effectually separated from the bakehouse by a partition, extending from the floor to the ceiling; and

(*b*) Has an external glazed window of at least nine superficial feet in area, of which, at the least four-and-a-half superficial feet are made to open for ventilation.

*Report of Medical Officer.*—The medical officer of health of every district council shall, in his annual report to them, report specifically on the administration of this Act in workshops and workplaces, and he shall send a copy of his annual report, or so much of it as deals with this subject to the Secretary of State (section 132). Where any woman, young person, or child is employed in a workshop in which no abstract of this Act is affixed as by this Act required, and the medical officer of the district council becomes aware thereof, he shall forthwith give written notice thereof to the inspector for the district.

Sale of Horseflesh for Human Food.

By section 1 of the Sale of Horseflesh Act, 1889, it is enacted that no person shall sell, offer, expose, or keep for sale any horseflesh for human food, elsewhere than in a shop, stall, or place over or upon which there shall be at all times painted, posted, or placed in legible characters of not less than four inches in length, and in a conspicuous position, and so as to be visible throughout the whole time, whether by night or day, during which such horseflesh is being offered or exposed for sale, words indicating that horseflesh is sold there. For the purposes of this Act "horseflesh" shall include the flesh of asses and mules, and shall mean horseflesh, cooked or uncooked, alone or accompanied by or mixed with any other substance.

Section 2 enacts that no person shall supply horseflesh for human food to any purchaser who has asked to be supplied with some meat other than horseflesh, or with some compound article of food which is not ordinarily made of horseflesh.

Any medical officer of health or inspector of nuisances or other officer of a local authority acting on the instructions of such authority or appointed by such authority for the purposes of this Act may at all reasonable times inspect and examine any meat which he has reason to believe to be horseflesh, exposed for sale or deposited for the purpose of sale, or of preparation for sale, and intended for human food, in any place other than such shop, stall, or place as aforesaid, and if such meat appears to him to be horseflesh he may seize and carry away or cause to be seized and carried away the same, in order to have the same dealt with by a justice (section 3).

On complaint made on oath by a medical officer of health or inspector of nuisances, or other officer of a local authority, any justice may grant a warrant to any such officer to enter any building, or part of a building other than such shop, stall, or place as aforesaid, in which such officer has reason for believing that there is kept or concealed any horseflesh which is intended for sale, or for preparation for sale for human food, contrary to the provisions of this Act; and to search for, seize, and carry away or cause to be seized and carried away any meat that appears to such officer to be such horseflesh, in order to have the same dealt with by a justice (Section 4).

Adulteration of Food.

Under section 13 of the Food and Drugs Act, 1875, any medical officer of health, inspector of nuisances, etc., may procure any sample of food or drugs, and if he suspect the same to have been sold to him contrary to any provision of this Act, shall submit the same to be analysed by the analyst of the district or place for which he acts. Under section 3 of the Food and Drugs Act, 1879, any medical officer of health, etc., may procure at the place of delivery any sample of any milk in course of delivery to the purchaser or consignee in pursuance of any contract for the sale to such purchaser or consignee of such milk; and such officer, if he suspect the same to have been sold contrary to any of the provisions of the Act of 1875, shall submit the same to be analysed. Under section 8 of the Margarine Act, 1887, a medical officer of health is empowered to take samples of margarine for analysis. Under section 14 of the Food and Drugs Act, 1899, the provisions of sections 3 and 4 of the Sale of Food and Drugs Act 1879 (relating to the taking of samples of milk in course of delivery), shall apply to every other article of food: provided that no samples shall be taken under this section except upon the request or with the consent of the purchaser or consignee.

Under section 14 of the Act of 1875 the person purchasing any article with the intention of submitting the same to analysis shall, after the purchase shall have been completed, forthwith notify to the seller or his agent selling the article his intention to have the same analysed by the public analyst, and shall divide the article into three parts, to be then and there separated, and each part to be marked and sealed or fastened up in such manner as its nature will permit, and shall, if required to do so, deliver one of the parts to the seller or his agent. He shall afterwards retain one of the said parts for future comparison and submit the third part, if he deems it right to have the article analysed, to the analyst.

